# Persistence of Pathogenic and Non-Pathogenic *Escherichia coli* Strains in Various Tropical Agricultural Soils of India

**DOI:** 10.1371/journal.pone.0130038

**Published:** 2015-06-23

**Authors:** S. Naganandhini, Z. John Kennedy, M. Uyttendaele, D. Balachandar

**Affiliations:** 1 Department of Agricultural Microbiology, Tamil Nadu Agricultural University, Coimbatore 641003, India; 2 Post Harvest Technology Centre, Tamil Nadu Agricultural University, Coimbatore 641003, India; 3 Laboratory of Food Microbiology and Food Preservation, Department of Food Safety and Food Quality, Faculty of Bioscience Engineering, Ghent University, B-9000 Ghent, Belgium; Cornell University, UNITED STATES

## Abstract

The persistence of Shiga-like toxin producing *E*. *coli* (STEC) strains in the agricultural soil creates serious threat to human health through fresh vegetables growing on them. However, the survival of STEC strains in Indian tropical soils is not yet understood thoroughly. Additionally how the survival of STEC strain in soil diverges with non-pathogenic and genetically modified *E*. *coli* strains is also not yet assessed. Hence in the present study, the survival pattern of STEC strain (O157-TNAU) was compared with non-pathogenic (MTCC433) and genetically modified (DH5α) strains on different tropical agricultural soils and on a vegetable growing medium, cocopeat under controlled condition. The survival pattern clearly discriminated DH5α from MTCC433 and O157-TNAU, which had shorter life (40 days) than those compared (60 days). Similarly, among the soils assessed, the red laterite and tropical latosol supported longer survival of O157-TNAU and MTCC433 as compared to wetland and black cotton soils. In cocopeat, O157 recorded significantly longer survival than other two strains. The survival data were successfully analyzed using Double-Weibull model and the modeling parameters were correlated with soil physico-chemical and biological properties using principal component analysis (PCA). The PCA of all the three strains revealed that pH, microbial biomass carbon, dehydrogenase activity and available N and P contents of the soil decided the survival of *E*. *coli* strains in those soils and cocopeat. The present research work suggests that the survival of O157 differs in tropical Indian soils due to varied physico-chemical and biological properties and the survival is much shorter than those reported in temperate soils. As the survival pattern of non-pathogenic strain, MTCC433 is similar to O157-TNAU in tropical soils, the former can be used as safe model organism for open field studies.

## Introduction

Shiga-like toxin producing *Escherichia coli* (STEC) strains are considered as an important food-borne pathogen [1]. STEC strains produce Shiga-like toxins (Stx1 and Stx2) and associated virulent factors such as intimin and enterohaemolysin [2]. Due to these, they can cause haemorrhagic colitis and haemolytic-uremic syndrome to human [3, 4]. STEC are common survivors in the ruminants’ intestine and can be transmitted to human through unprocessed foods [5, 6]. Elderly people and young children are most sensitive to STEC mediated food-borne infections. Though several serogroups (O26, O55, O91, O103, O111 or O145) are associated with human diseases, *E*. *coli* O157:H7 is the most frequent serotype involved in the worldwide outbreaks.

Contaminated foods such as dairy products, undercooked minced beef and raw fruit and vegetables from contaminated field are the important sources for O157:H7 based illness [7, 8]. Cattle and other farm animals are the main reservoir for O157:H7 and their direct or indirect contract with agricultural soils associated to an increasing number of infections [5, 6]. *E*. *coli* O157:H7 can survive in soil and animal based manures for long period (ranged from 25 to more than 365 days) [9, 10] and low cell load (10–500 per g) is enough to cause the infection to human [11]. Partially decomposed manures, animal slurries, slaughterhouse wastes and human sewage are the potential source of contamination of arable agricultural lands [12–14].

Several studies have focused on the survival of *E*. *coli* O157:H7 in soil [15–21]. The survival of *E*. *coli* O157:H7 depends on the soil type [17, 22–24], texture [25], physico-chemical properties and indigenous soil microbiome [26, 27] and land use patterns [28, 29]. The soil organic carbon (SOC) and organic nitrogen are the major drivers reported for long survival of *E*. *coli* O157:H7 in organically manured soils [17]. Likewise, Van Elsas et al. [30] pointed out that soil microbial community shift due to fumigation significantly influenced the survival of *E*. *coli* O157:H7. High moisture content of the soil (17–32%) hasten the decay of O157:H7 as compared to low moisture levels (2–8%) [23]. Yao et al. [31] and van Elsas et al. [32] showed *E*. *coli* O157:H7 survival was affected by indigenous microorganisms in soil. The difference in survival of *E*. *coli* O157:H7 in soil due to various factors indicate the difference in the potential risk of pathogen contamination from soil environment. Hence, more knowledge on survival of *E*. *coli* in soils will facilitate to reduce the risk of pathogen contamination and avoiding infection from the pathogen. Under Indian perspective, no study has been so far done on the survival of *E*. *coli* O157:H7 in Indian agricultural soils, though the climatic and soil physico-chemical properties of Indian sub-continent favour the *E*. *coli* existence.

*E*. *coli* O157:H7 can survive in organic manures with virulence up to 70 days [33]. Composting process generates sufficient heat due to microbial actions may kill the pathogens including O157:H7. Obviously, the well-decomposed compost should be free from pathogens. However, the environmental conditions, temporal variability of composting materials and improper handling of compost may not kill all the pathogens. When such manures are spread on the lands can lead to pathogen entry to the food chain. Root and leafy vegetables, especially, raw eaten vegetables have the risk of contamination from such manure application to soil. The water used for irrigation can also spread the pathogen in the agricultural soils. Such introduced *E*. *coli* varied in their survival pattern depending upon the soil type, clay contents, soil organic carbon content and so on. Genetically modified strains by recombinant DNA techniques have the potential to interfere with native microbial populations and their processes in soil. The genetically modified *E*. *coli* strain had less competitive survival than the wild strain in soil and its survival potential depends on the inoculum level and soil microflora [34]. Hence, in the present work, the survival of *E*. *coli* O157:H7 was compared with non-pathogenic and genetically modified *E*. *coli* strains in four Indian agricultural soils and an organic medium for nursery (coconut coirpith waste called cocopeat) suitable for vegetables cultivation. The incubation studies were conducted under controlled lab condition to address the following key questions: 1. How-long O157 can survive in Indian agricultural soils, 2. Is there any difference in survival rates between pathogenic *E*. *coli* and non-pathogenic *E*. *coli* in agricultural soils.

## Materials and Methods

### *E*. *coli* strains

*E*. *coli* O157, isolated, characterized for presence of *stx1* and *stx2* (Shiga-like toxins producing genes), *eae* (intimin producing gene) from contaminated irrigation water at Tamil Nadu Agricultural University, Coimbatore (designated as O157-TNAU) was used for this study. This isolate showed susceptibility to ampicillin antibiotic. The non-pathogenic strains, MTCC433 (equivalent to ATCC15223/ DSM1058/ NCIB9552) and DH5α (genetically engineered strain) were used for this study. The genotypic characters of all the three strains used in the experiment were presented as Table 1.

**Table 1:** *E*. *coli* strains used in the present study.

*E*.*coli* strains	Genotype	Reference
*E*.*coli* DH5α	*F^-^, endA1, hsdR17(r_K_^-^ m_K_^+^), glnV44, thi-1deoR, gyrA96, recA1, relA1, supE44, Δ(lacZYA-argF) U169, λ-, (Φ80dlacZΔM15), nupG*, Non-pathogenic strain developed in the laboratory for routine cloning applications.	[71]
*E*.*coli* MTCC433	Non-pathogenic strain isolated from human intestine. Inducible for β-galactosidase	[72]
*E*.*coli* 0157-TNAU	O157 strain isolated from irrigation water of TNAU farm (Storage tank water) showed positive for toxin genes *stx1*, *stx2* and 0157 *uid*A allele.	Present study

### Green fluorescent protein (GFP) labeled *E*. *coli* strains

**Fig 1:**
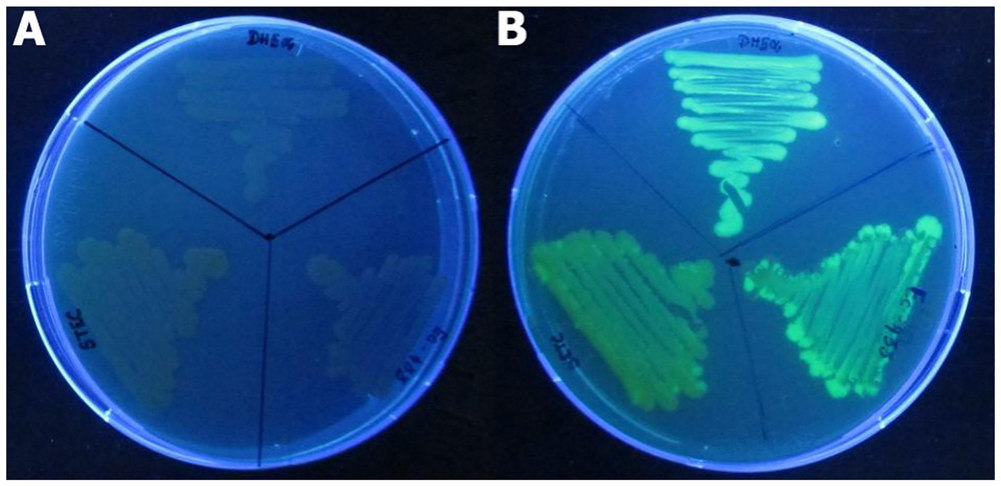
Wild (A) and EGFP tagged (B) *E*. *coli* strains used in the present study. The pGreenTIR conferring ampicillin resistance and enhanced green fluorescence under UV was transformed to all the three strains, viz., DH5α, MTCC433 and O157-TNAU.

For easy detection in soil under controlled condition, all the three strains were introduced with plasmid pGreenTIR [35], a derivate of pUC18 in which mutated *gfp* gene (containing a double F65L S65T amino acid change that increases green fluorescent protein (GFP) stability and fluorescence) and ampicillin resistance gene are expressed. This plasmid also contains an improved translation initiation region for prokaryotes, including the translational enhancer and the Shine–Dalgarno regions of phage T7 gene 10, so that synthesis of GFP is enhanced. All the three *E*. *coli* strains grown in Luria Birtani (LB) medium (HiMedia, India) were transformed with pGreenTIR plasmid by following standard CaCl_2_-method with selection for ampicillin resistance (100 μg/ml) and GFP production under UV light (Fig 1). Virulence profiles (*stx1* and *stx2* genes) of the three strains with and without pGreenTIR were characterized by PCR as primers and conditions described by Cebula et al. [36]. Additionally, the *uidA* gene, responsible for β glucuronidase activity specific to *E*. *coli* was also confirmed by PCR [36]. Further, genome fingerprints of each strain with and without plasmid were characterized by BOX-A1R-based repetitive extragenic palindromic-PCR (BOX-PCR) using BOX-A1R primer [37] as previously reported by Urzì et al. [38].

### Soil and organic plant growth medium

Four different soils viz., wetland soil, red lateritic soil, black cotton soil and tropical latosol soil were collected from different agro-ecological zones of Tamil Nadu, India in sterile containers. Samples were collected from Tamil Nadu Agricultural University research farms and three independent samples were maintained per soil. Soil samples from 0–30 cm collected from 10 different locations of a field, pooled, removed from stones and stubbles were powdered, packed in water and air tight plastic bags and stored at 4°C for all the analyses. The cocopeat, organic medium for vegetable nursery was collected from local firm. Soil pH and electrical conductivity (EC) were estimated with a glass electrode using a soil to water ratio of 1:1. Soil organic carbon was determined by dichromate oxidation [39]. Soil available N was extracted with 2 M KCl for 1 h and determined by Kjeldahl method [40]. Available P was extracted with Olsen reagent [0.5 M NaHCO_3_ (pH 8.5)] at soil-extractant ratio of 1:20, shaken for 30 min and quantified by molybdenum—blue colorimetry [41]. Available K was extracted with neutral normal ammonium acetate (pH 7.0), shaken for 25 min and measured by flame photometry [42]. The microbial biomass carbon (MBC) was quantified by fumigation extraction method [43]. Dehydrogenase (EC 1.1.1.1) was determined by the reduction of triphenyltetrazolium chloride to triphenylformazan (TPF) and reported as μg of TPF released per g soil per day [44]. The physico-chemical properties of soil samples and cocopeat were presented as Tables 2 and 3.

**Table 2:** Physico-chemical properties of soils and geographical coordinates of the soil collection sites used in the present study.

Physico-chemical properties	Wetland soil	Red lateritic soil	Black cotton soil	Tropical latosol
Texture	Clay-loam	Clay	Loam	Loam
pH	8.74 (±0.04)^a^	5.97 (±0.03)^d^	8.42 (±0.04)^b^	6.05 (± 0.01)^c^
EC (dSm^-1^)	0.23 (±0.01)^a^	0.24 (±0.01)^a^	0.31 (±0.03)^a^	0.56 (±0.05)^a^
Soil organic carbon (%)	0.45 (±0.07)^bc^	0.65 (±0.12)^b^	0.26 (±0.20)^c^	1.53 (±0.13)^a^
Available N (Kg/ha)	223.07 (±1.87)^b^	304.27 (±4.94)^a^	233.13 (±3.37)^b^	325.73 (±7.64)^a^
Available P (Kg/ha)	11.07 (±0.70)^bc^	12.99 (±0.71)^b^	8.53 (±0.71)^c^	18.02 (±0.30)^a^
Available K (Kg/ha)	273.33 (±16.67)^b^	270.00 (±5.77)^b^	376.67 (±6.67)^a^	273.33 (±6.67)^b^
Microbial biomass carbon (μg/g)	3454.8 (±32.6)^d^	9957.0 (±280.3)^a^	5744.4 (±628.1)^c^	7968.9 (±576.4)^b^
Dehydrogenase (μg TPF released/ g soil/day)	17.71 (±0.50)^d^	57.51 (±6.46)^a^	22.48 (±6.73)^c^	41.38 (±1.38)^b^
Geographical coordinates of sampling site	11.12°N latitude; 76.99°E longitude; 426 m altitude	10.4°N latitude; 78.82°E longitude; 102 m altitude	9.17°N latitude; 77.87°E longitude; 106 m altitude	11.41°N latitude; 76.70°E longitude; 2242 m altitude

Values are mean (± standard error) (n = 5) and values followed by the same letter in each row are not significantly different from each other as determined by DMRT (*p* ≤ 0.05). EC—Electrical conductivity; TPF—triphenylformazan.

**Table 3:** Physico-chemical characteristics of cocopeat used in the present study.

Physico-chemical properties	Value^a^
pH	5.96 (±0.01)
EC (dSm^-1^)	4.74 (±0.33)
Organic carbon (%)	27.62 (±0.95)
N (%)	0.30 (±0.02)
P (%)	0.03 (±0.00)
K (%)	0.88 (±0.04)
Microbial biomass carbon (μg/g)	3145.2 (±139.2)
Dehydrogenase (μg TPF released/ g /day)	5.24 (±1.20)

^a^ Value represents mean (± standard error) (n = 5). EC—Electrical conductivity; TPF—triphenylformazan.

### Inoculum preparation

Each GFP-labeled *E*. *coli* strain was grown overnight at 37°C on Luria Bertani plates [45] containing ampicillin (100 μg/ml). Then, each strain was cultivated in 1 l of LB broth supplemented with ampicillin (100 μg/ml) at 37°C for 24 h. All the cultures reached a final concentration of approx. 10^11^ colony forming units (cfu) per ml. The bacteria were pelletized by centrifugation at 5000 g for 20 min at room temperature and cell pellets were re-suspended in 100 ml of phosphate buffered saline (PBS) and centrifuged. This operation was repeated and afterwards the cell pellets were re-suspended in 100 ml of PBS.

### Soil incubation study

Unsterilized experimental soils and cocopeat were adjusted nearly 50% moisture holding capacity with sterile distilled water. The *E*. *coli* cell suspension prepared as inoculum was thoroughly mixed with soils and cocopeat with a final concentration of 10^7^ cfu per g in a plastic bag. From this, a quantity of 500 g of the inoculated soil was transferred to a perforated sterile containers (HiMedia, India) for air exchange. The same amount of non-inoculated soil added with deionized water instead of cell suspension was maintained as control. The moisture per cent was maintained during the course of experiment by adding additional sterile deionized water weekly to obtain original weight of the container. Three replicates were maintained per sample. The inoculation was done following all safety procedures in Class-II Biological safety cabinet (Nuaire, USA) and the containers were incubated at temperature-controlled incubator (Lab Companion, USA) at 30°C.

### Sampling and enumeration of *E*. *coli*

The population of each sample measured on the day of inoculation (0 day) and subsequently at 5 days intervals for a period of 45 days. A quantity of 10 g of homogenized soil sample was withdrawn from the container at each sampling time. *E*. *coli* cells from each sample were extracted by 0.1% peptone buffer (HiMedia, India) and the resulting soil suspension was subjected to a 10-fold serial dilutions and enumerated by plating on LB agar supplemented with ampicillin (100 μg/ml). The fluorescent colonies visualized through UV-illuminator were counted and expressed as cfu per g dry weight of sample. The detection limit of the plating technique is about 100 cfu per g and sampling was stopped after the day at which one of the strains was at below detectable limit. For safety issues, the sampling, plating, colony counting were performed in Class-II Biological safety cabinet (Nuaire, USA) and proper disposal procedures were followed for both colony containing plates and inoculated soils.

### Statistical analysis and modeling of bacterial survival

All the data were subjected to statistical analysis with software, Microsoft Excel for Windows 2007 add-ins with XLSTAT version 2010.5.05 [46]. Statistically significant differences between soil samples and *E*. *coli* strains were analyzed using one-way analysis of variance (ANOVA) and Duncan’s multiple range test (DMRT) at 5% significance level.

All the microbial counts (cfu per g) were log transformed (log_10_ cfu per g). The log-transformed data were then fitted for modeling of survival of *E*. *coli* strains using GInaFIT version 1.6 [47]. This freeware tool enables the generation of statistical measures and parameter values of the survivor curves [47]. The double Weibull survival model [48] was constructed based on the hypothesis that the population is composed of two subpopulations differing in their capability on resistance to stress and deactivation kinetics of both subpopulations follows a Weibull distribution. The size of the surviving population can be calculated using following equation.

$$N_{\left(t\right)}=\frac{N_{0}}{1+10^{\alpha }}\left[10^{-{\left(\frac{t}{\delta 1}\right)}^{p}+\alpha }+10^{-{\left(\frac{t}{\delta 2}\right)}^{p}}\right]$$

In the equation, N is the number of survivors (cfu per g), N_0_ is the initial inoculum size (cfu per g), t is the time (days), *p* is the shape parameter (dimensionless, when *p*>1, convex curve is observed; when *p*<1, concave curve is observed, when *p* = 1, a linear curve is observed), δ_1_ is the time for the first decimal reduction of subpopulation 1 (days), δ_2_ is the time for first decimal reduction of the second subpopulation (days) and α is the log_10_ of the ratio of the fraction of more sensitive subpopulation to the fraction of less sensitive subpopulation at time zero. The *ttd* (time needed to reach detection limit, 100 cfu per g soil) was calculated using GInaFiT.

To reveal the similarities and differences between samples and to assess the relationships between the observed soil variables and *E*. *coli* survival, principal component analysis (PCA) [49] was performed on all the data. The number of variables was reduced by excluding those explained to less than 50% by the significant components in PCA.

## Results

### Stability of GFP-tagged *E*. *coli* strains

**Fig 2:**
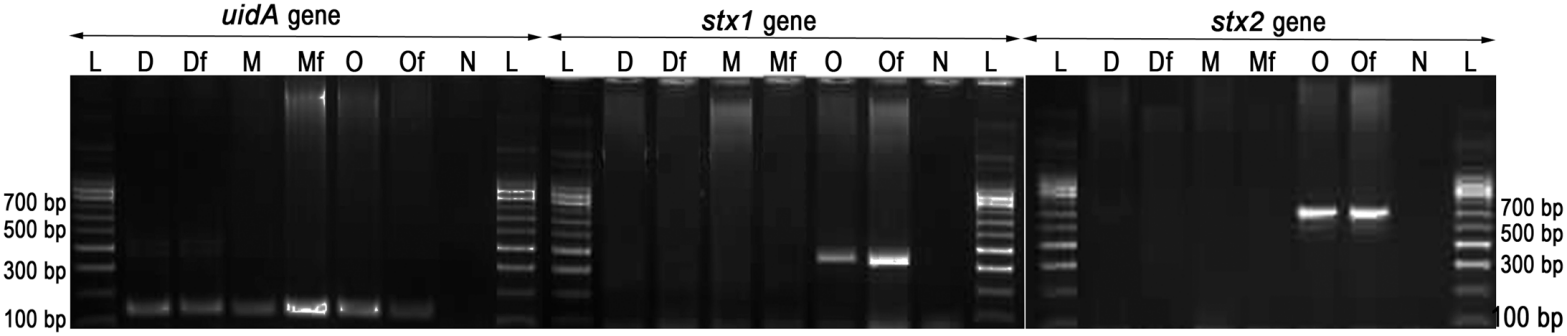
PCR confirmation of *E*. *coli* strains (*E*. *coli* specific *uidA* gene) and virulence genes (*stx1* and *stx2*). L—100 bp DNA ladder; D—DH5α; Df—DH5α with pGreenTIR (Fluorescent); M—MTCC433; Mf—MTCC433 with pGreenTIR (Fluorescent); O— O157-TNAU; Of— O157-TNAU with pGreenTIR (Fluorescent); N—Negative control.

**Fig 3:**
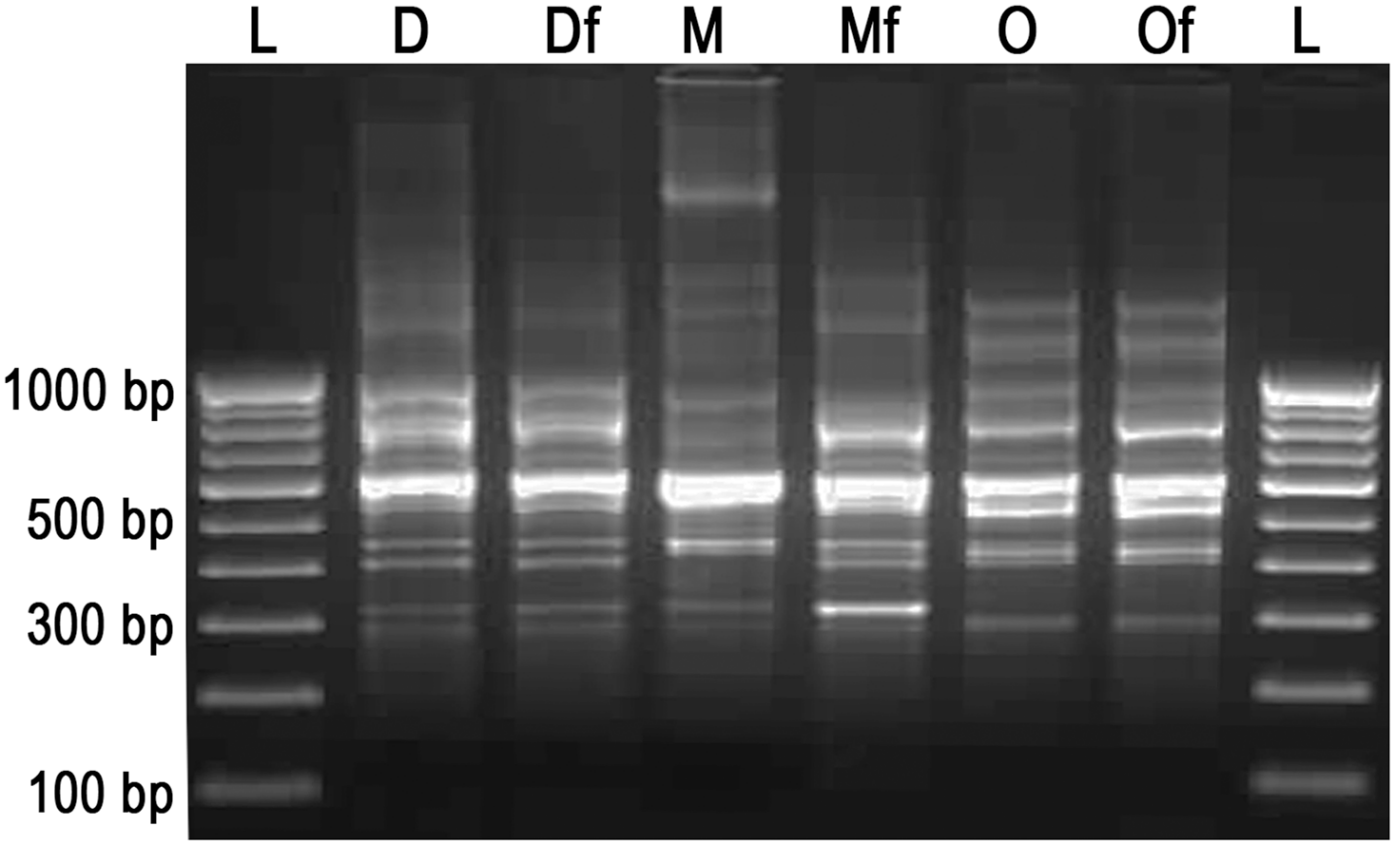
BOX-PCR fingerprints of wild and GFP-tagged strains of *E*. *coli*. L—100 bp DNA ladder; D—DH5α; Df—DH5α with pGreenTIR (Fluorescent); M—MTCC433; Mf—MTCC433 with pGreenTIR (Fluorescent); O— O157-TNAU; Of— O157-TNAU with pGreenTIR (Fluorescent).

The stability of plasmid (pGreenTIR) in all the three *E*. *coli* strains and constitutive expression of GFP were performed by repeated sub-culturing and also by long incubation (even after 6 months) in LB broth containing ampicillin (100 μg/ml) at 37°C. In all the cases, the strains showed ampicillin resistance and stable GFP expression (Fig 1). The PCR targeting virulent genes (*stx1* and *stx2*) of *E*. *coli* strains with or without pGreenTIR showed same pattern of amplification for both the genes. A 348-bp amplicon for *stx1* and 585-bp amplicon for *stx2* were observed in O157-TNAU strains with and without pGreenTIR (Fig 2). *E*. *coli* species specific *uidA*-PCR was performed for all the three strains with and without plasmid. The results also confirmed the presence of 148-bp amplicon of partial *uidA* gene for both wild and GFP-tagged strains (Fig 2). The genome fingerprints between the wild strain and its corresponding GFP-tagged strain did not show any difference in terms of banding pattern (Fig 3). All the three strains capable of producing 9–10 bands ranged 300–900 bp sized with the difference in the banding patterns. These results showed that no genetic change was observed in any of the three strains due to introduction of GFP-plasmid.

### Survival of *E*. *coli* strains in soils and cocopeat

**Fig 4:**
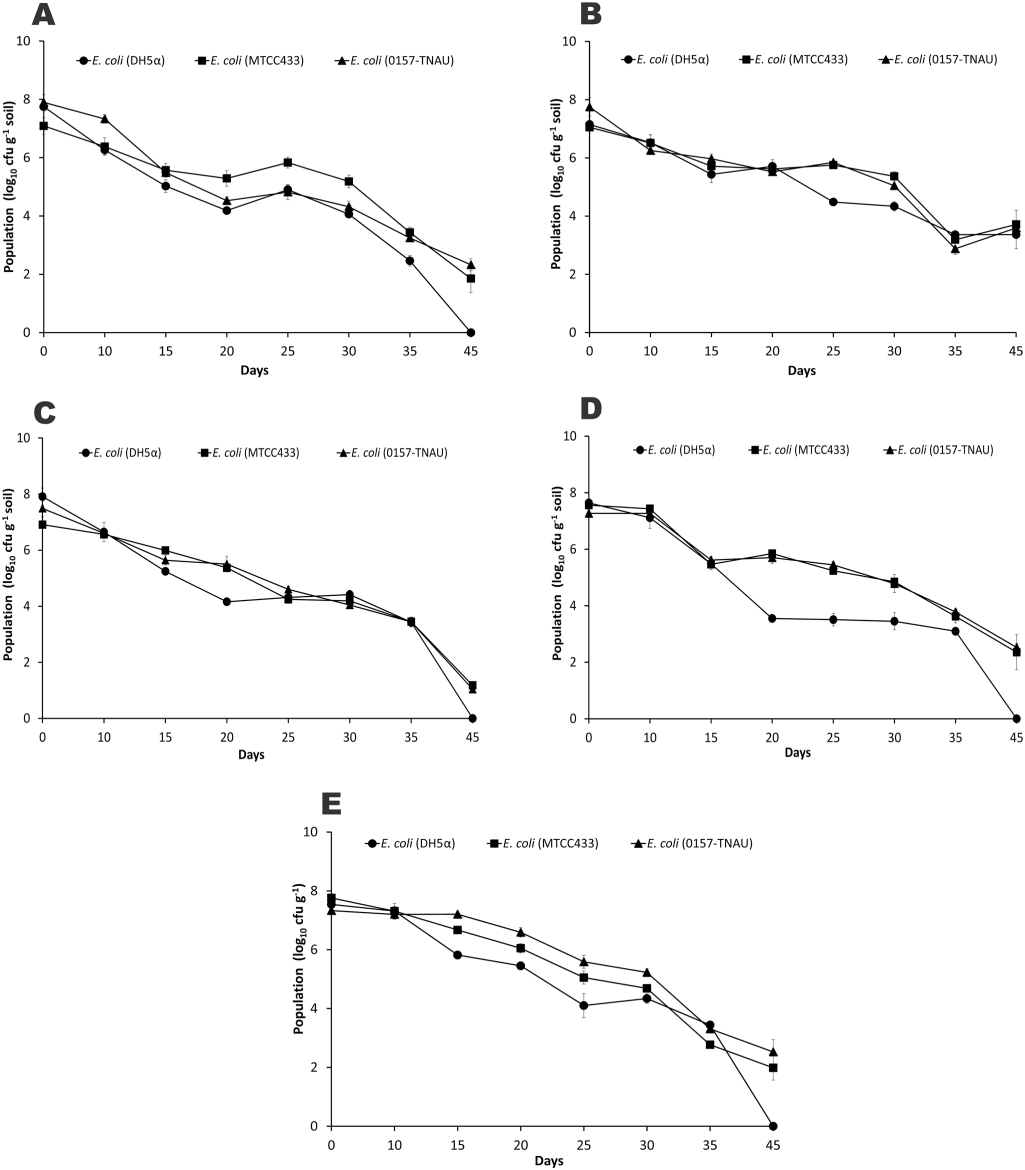
Survival of *E*. *coli* strains in different tropical soils and cocopeat under controlled condition. A—Wetland soil; B—Red lateritic soil; C—Black cotton soil; D—Tropical latosol; E—Cocopeat. Means of three replicate values plotted and errors bars indicate the standard error.

The cell counts of all the inoculated *E*. *coli* strains decreased gradually till the detection limit of the plating method (45 days post-inoculation). Among the three strains, DH5α perished quicker than MTCC433 and O157-TNAU (Fig 4). Except red laterite soil, all other samples had DH5α population at below detectable limit (100 cfu per g of soil) at 45^th^ day. The MTCC433 and O157-TNAU exhibited uniform pattern of decay in all the five samples. Among the four soils tested, red laterite and tropical latosol reported to have slow decline of cell counts of *E*. *coli* (especially MTCC433 and O157-TNAU) (Fig 4A and 4D). These strains’ survival rates were differed in cocopeat, of which MTCC433 had less survival rate than O157 (Fig 4C and 4E).

**Fig 5:**
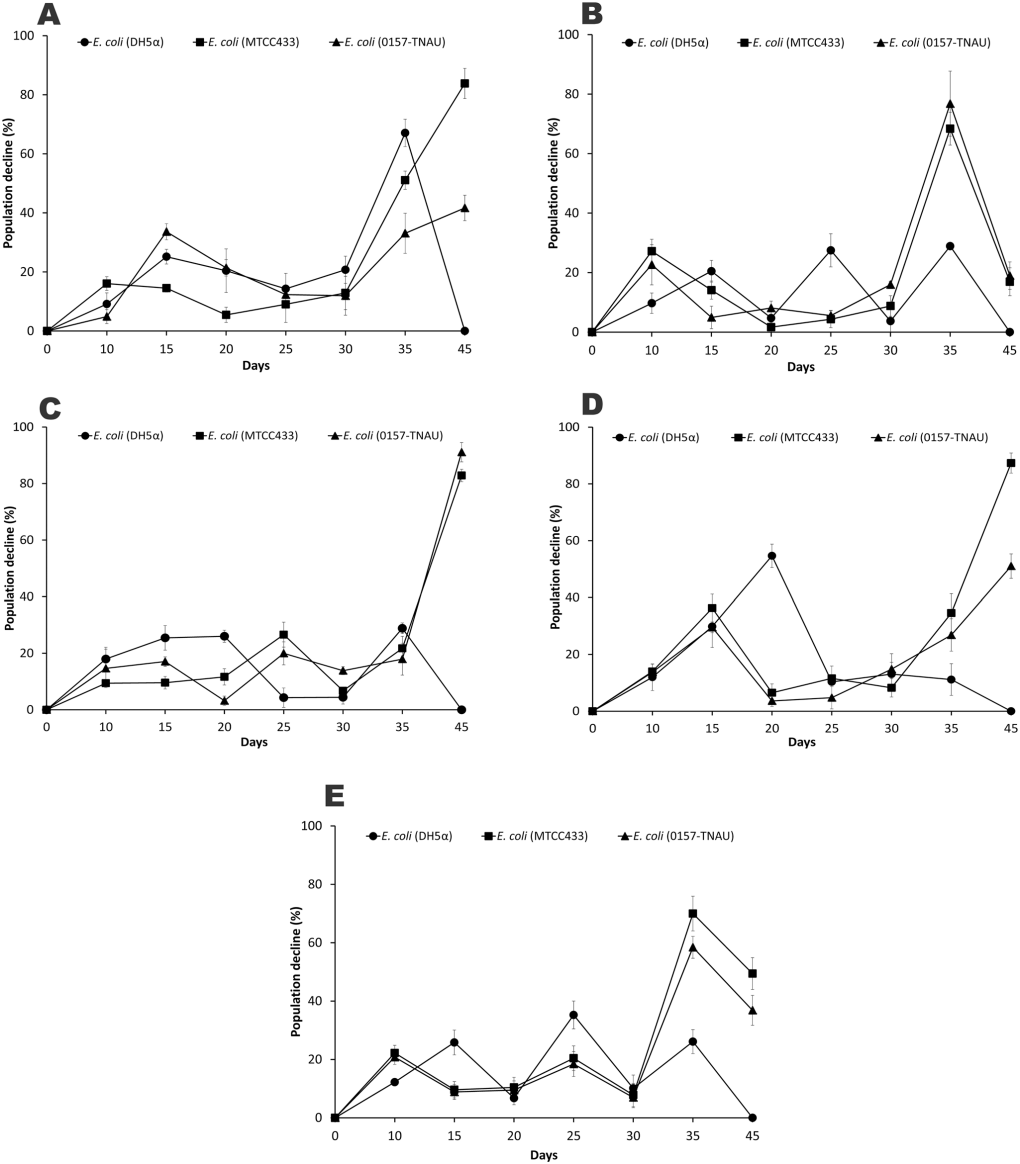
Per cent population decline of *E*. *coli* strains in different tropical soils and cocopeat under controlled condition. A—Wetland soil; B—Red lateritic soil; C—Black cotton soil; D—Tropical latosol; E—Cocopeat. Means of three replicate values plotted and errors bars indicate the standard error.

When calculating the population declining per cent, all the strains and soils showed a mean decline of about 17–20% up to 30 days of incubation and suddenly increased to 60–80% in later stages (Fig 5). In this observation also, DH5α differed from MTCC433 and O157-TNAU. Among the soils and medium assessed, cocopeat showed a near uniform and gradual decline (Fig 5E) compared to other soils, while the declining per cent was most erratic in tropical Latosol (S4, Fig 5D).

Statistical measures of fits and parameter values of the fitted curves for the survival of all the three *E*. *coli* strains in four different soil samples and cocopeat according to Double Weibull model are presented in Table 4. All the three *E*. *coli* strains had significantly shorter *t*_4D_ (about 10 days) in wetland soil (S1), black cotton soil (S3) and cocopeat (S5) than red laterite soil (S2) and tropical latisol (S4). Among the strains, there is no significant difference was noticed for *t*_4D_. The δ1 (days for first decimal reduction of sub-population) parameter value was significantly higher in MTCC433 and O157-TNAU for S2 soil (15.54 and 14.65 respectively) than other soils and strains. Likewise, the lowest δ1 was reported for all the three strains in S1 and S3 soils (ranged from 3.3–4.9). The mean δ1 for cocopeat for all the three strains was about 8.0. The δ2, days for second decimal reduction of sub-population, also followed the same trend as that of δ1, however with relatively higher values (about 10–15 days). The significance of δ2 among the soil samples was more than δ1. Likewise, a significant difference among the soil types and strains of *E*. *coli* was observed for shape parameter (*p*) also. When considering the rate parameters of Double Weibull model (δ1, δ2 and *p*), it is clear that all five soils showed significantly different survival curves for the three different strains of *E*. *coli*. In terms of E. coli survival rate, the modeling parameters exhibited that S2 and S4 were in one group, while S1. S3 and S5 formed another group. With reference to strains, DH5α responded different than MTCC433 and O157-TNAU. This trend was also confirmed by *t*_4D_ values.

**Table 4:** Statistical measures and parameter values of the fitted model describing the survival of different *Escherichia coli* strains in different soils and cocopeat under controlled condition according to the Double Weibull model.

Soil	RSME	Adj R^2^	t_4D_	N_0_	α	δ1	δ2	*p*
DH5α								
S1	0.18	0.99	26.70^e^	7.75^a^	2.82^c^	3.38^d^	24.92^f^	2.69^bc^
S2	0.23	0.97	39.00^a^	7.15^a^	2.22^c^	13.17^b^	36.54^a^	1.54^d^
S3	0.46	0.97	32.55^bc^	7.91^a^	2.70^c^	4.07^d^	24.62^f^	3.07^ab^
S4	0.46	0.98	38.45^a^	7.64^a^	4.07^b^	9.30^c^	30.42^bc^	1.34^d^
S5	0.35	0.94	27.00^e^	7.54^a^	2.88^c^	8.34^c^	25.83^f^	3.11^ab^
MTCC433								
S1	0.26	0.98	29.00d^e^	7.09^a^	1.38^e^	3.80^d^	22.41^g^	3.55^a^
S2	0.40	0.96	39.25^a^	7.05^a^	4.84^b^	15.54^a^	32.54^b^	0.96^e^
S3	0.27	0.98	29.95^cd^	6.91^a^	1.68^e^	4.56^d^	24.77^f^	3.11^ab^
S4	0.29	0.99	39.25^a^	7.56^a^	2.80^c^	9.50^c^	29.41^cd^	0.81^e^
S5	0.32	0.97	27.30^e^	7.76^a^	7.29^a^	8.43^c^	28.77^cd^	1.21d^e^
O157-^TNAU^								
S1	0.45	0.95	30.45^cd^	7.90^a^	3.61^d^	3.47^d^	12.45^h^	1.07d^e^
S2	0.40	0.93	38.10^a^	7.75^a^	1.45^e^	14.65^ab^	32.84^b^	2.28^c^
S3	0.11	1.00	34.50^b^	7.49^a^	1.89^e^	4.94^d^	14.45^h^	2.90^b^
S4	0.28	0.98	38.65^a^	7.27^a^	2.48^c^	8.58^c^	26.55d^e^	2.89^b^
S5	0.45	0.96	27.00^e^	7.33^a^	6.74^a^	8.19^c^	24.48^f^	1.17d^e^

S1—Wetland soil; S2—Red lateritic soil; S3—Black cotton soil; S4—Tropical latosol; S5—Cocopeat. Values are means ± standard error of three replicates and values followed by the same letter in each column are not significantly different from each other as determined by DMRT (*p* ≤ 0.05). RMSE, root mean sum of squared error; AdjR^2^, adjusted R^2^; t_4D_, time (days) to attain a 4 log reduction; N_0_, initial cell count (log CFU g^-1^); α, parameter that relates the fraction of the first subpopulation to the second subpopulation; δ1, time (days) for first decimal reduction of subpopulation 1; δ2, time (days) for first decimal reduction of subpopulation 2; *p*, shape parameter.

The time to reach the detection limit (*ttd*, 2.0 log_10_ cfu per gram soil) calculated according to Double Weibull model showed significant difference among the soils (Table 5). The model derived *ttd* for all the strains was significantly shorter in cocopeat (39–40 days; 69 days for O157), Black cotton soil (40–46 days) and wetland soil (41–47 days) than red lateritic (58–68 days) and tropical latisol (60–62 days) soils. Among the three strains, O157-TNAU showed significantly varied *ttd* among the soil types, while MTCC433 and DH5α showed more or less uniform trend.

**Table 5:** Time to reach the detection limit (*tdd*) of the plate count method (2 log_10_ cfu per g) for *E*. *coli* strains in different soils and cocopeat under controlled condition according to the Double Weibull model.

Soils	Time to reach detection limit (*ttd*) of plate count method		
	DH5α	MTCC433	O157-TNAU
S1	41.77 (± 0.16)^b^	44.64 (± 0.02)^b^	47.83 (± 0.03)^c^
S2	58.84 (± 0.02)^a^	60.13 (± 0.02)^a^	68.13 (± 0.03)^a^
S3	40.64 (± 0.03)^b^	41.05(± 0.06)^b^	42.89 (± 0.05)^d^
S4	41.52 (± 0.04)^b^	60.10 (± 0.06)^a^	62.02 (± 0.02)^b^
S5	41.50 (± 0.04)^b^	40.82 (± 0.12)^c^	69.75 (± 0.02)^a^

S1—Wetland soil; S2—Red lateritic soil; S3—Black cotton soil; S4—Tropical latosol; S5—Cocopeat. Values are means ± standard error of three replicates and values followed by the same letter in each column are not significantly different from each other as determined by DMRT (*p* ≤ 0.05).

### Principal component analysis

**Fig 6:**
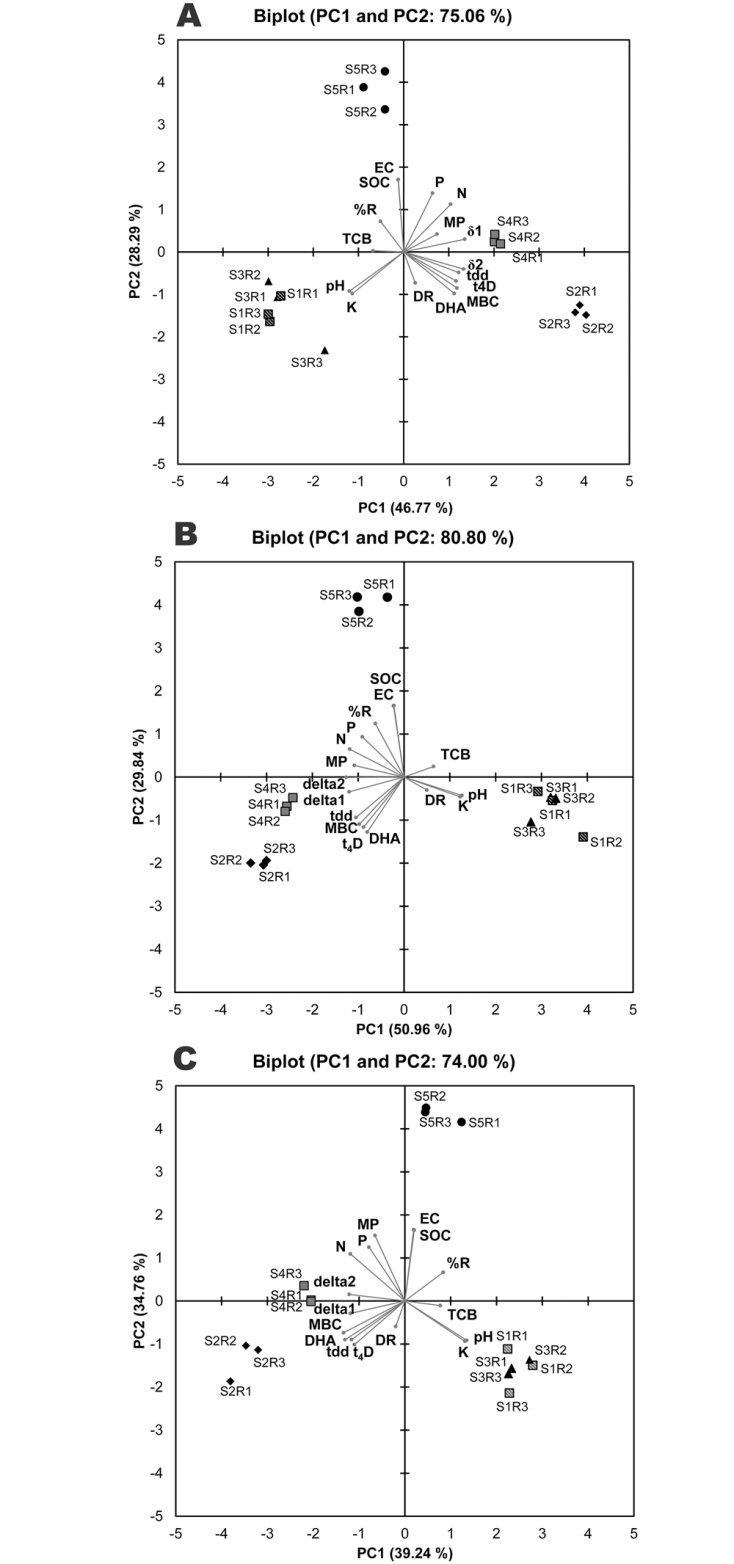
PCA biplot showing orthogonal positions of soils and cocopeat and assessed soil variables and survival modeling parameters of three *E*. *coli* strains. A—DH5α; B—MTCC433; C- O157-TNAU. S1—Wetland soil; S2—Red lateritic soil; S3—Black cotton soil; S4—Tropical latosol; S5—Cocopeat. Soil variables: pH; EC, electrical conductivity; N, P, K, available nutrients; SOC, soil organic carbon; MBC, microbial biomass carbon; DHA, dehydrogenase; TCB, total culturable bacterial count. *E*. *coli* survival parameters: %R, population decline per cent; MP, mean population reduction rate; DR, Decimal reduction rate. Survival modeling parameters: t_4D_, time (days) to attain a 4 log reduction; δ1, time (days) for first decimal reduction of subpopulation 1; δ2, time (days) for first decimal reduction of subpopulation 2; ttd, time to reach the detection limit.

The principal component analysis of assessed variables showed that first and second components explain 74 to 80% of total variance among the *E*. *coli* strains, of which PC1 contributes 39–46%, while PC2 adds another 28–34%. The bi-plots describing the orthogonal positions of soils and assessed variables explained by first two PCs are presented as Fig 6. For DH5α, the pH, microbial biomass-C, N, K, DHA, *ttd*, *t*_4D_, δ1, δ2 contributed nearly 80% of the total variability of PC1 (46.78%) (Fig 6A). However, the concentration of K and soil pH showed negative loading values while the other significant variable showed positive (S1 Table). In case of non-pathogenic *E*. *coli* strain MTCC433, pH, microbial biomass-C, DHA, N, P, K, *ttd*, *t*_4D_, δ1, δ2 added 91.85% of total variability of PC1 (50.96%) (Fig 6B). The *ttd*, *t*_4D_, δ1, δ2, MBC, DHA, N, P, K showed negative loading values with rest of the variables had positive loading values. The soil pH, MBC, DHA, available N and K, *ttd*, *t*_4D_, δ1, δ2 contributed nearly 85% of PC1 (39.24) in case of O157-TNAU strain (Fig 6C). The *ttd*, *t*_4D_, δ1, δ2, MBC, DHA and N had negative loading values (S1 Table). In all the three samples, soil organic carbon showed insignificant contribution to the PC variability.

The positions of soil samples in the bi-plot defined by first two PCs showed variability between DH5α and rest of the strains (MTCC433 and O157-TNAU) (Fig 6). Invariable to three *E*. *coli* strains, cocopeat (S5) is clearly discriminated from other soil samples. Among the soils, red lateritic and tropical latosol soils (S2 and S4) clustered tightly, while wetland and black cotton soils (S1 and S3) are indistinguishable in the bi-plots (Fig 6). With relation to the position of samples and the assessed soil variables, a considerable variation was observed between DH5α and rest of the strains (MTCC433 and O157-TNAU). The soil samples in which DH5α perished slowly (S2 and S4) were positioned in the right end of PC1 while the quickly perished soils (S1 and S3) were in the left end of PC. In case of MTCC433 and O157-TNAU, the trend was in opposite to DH5α. Irrespective to soils and *E*. *coli* strains, the three replicated samples are tightly clustered together.

## Discussion

In the present study, we have examined whether the survival pattern of O157:H7 differed among the tropical soils and vegetable growth medium and also differed from non-pathogenic *E*. *coli* strains. We followed the survival of *E*. *coli* strains in the soil in a similar way as performed in previous studies [18, 50, 51].

The survival of O157:H7 in soil and manure was assessed using marker strains of O157:H7 in past. Some authors used spontaneous mutants of O157:H7, devoid of *stx* genes showing rifampicin or nalidixic acid resistance for assessing the survival in soil [18, 52–54]. Oliveira et al. [19] used non-pathogenic strain of O157:H7 unable to produce virotoxins (Stx1 and Stx2), used for soil incubation studies and counted using O157 specific medium (Sorbitol MacConkey agar supplemented with cefixime and tellurite). For controlled incubation studies, the pathogenic strains were tagged with rifampicin and kanamycin resistant genes by trans-conjugation and monitored their survival [21, 55]. On the other hand, several researchers introduced stable plasmids capable of producing enhanced green fluorescent protein (pGFP or pGFPuv) to virulent strains [24, 50, 56, 57] or to the avirulant strains [51, 58] and used for soil incubation studies. Nevertheless, all the results are comparable for the survival of O157:H7 in manure or soil or manure amended soils. In another exclusive study made by Ma et al. [55] found that mutants of O157 with either *stx1* or *stx2* or both did not show any significant difference in the survival pattern. This result clearly suggests that the virulence genes did not play any direct role in the survival of this pathogen in soil. In the present study, we have used a GFP-Plasmid, pGreenTIR to tag the *E*. *coli* strains with ampicillin resistance and fluorescence under UV light. For O157 strain, we have not mutated the toxin producing genes, as the whole experiment was done under controlled conditions. This method found to be convenient for quantification of the *E*. *coli* cells from soils and the methodology and results found to be comparable to most of the earlier works [24, 57]. To reveal the genetic stability of *E*. *coli* strains, pulse field gel electrophoresis (PFGE) was performed in past for O157 strains [21, 57]. In present study, we have used BOX-PCR fingerprinting method, which is quicker and cheaper method and more discriminative than PFGE [59]. There is no significant difference in BOX-PCR based genome fingerprints found between the wild and GFP-tagged strains further confirmed that the introduction of marker plasmid did not cause genome integrity of *E*. *coli* strains.

The survival time for O157:H7 in soil ecosystem varied across the global observations. It was expected that the soils from temperate climatic condition would be different from tropical climate because of unique differences in soil properties, moisture condition, temperature regime and cropping pattern. The tropical soils had much shorter survival times (35–75 days: [18]; 21–28 days: [24, 60]; 103 days: [50]) than those from temperate climate (21 months: [61]; 120 days: [62]; 154–231 days: [63]; 300 days: [64]). In the present study, the survival time reported (40–60 days in all soil types) is well-fitted to the tropical soils. These results suggest that the biophysical conditions in the tropics might be more detrimental to *E*. *coli* O157:H7 than temperate conditions. Additionally, these results also revealed that even within tropical isothermal conditions, the survival time varies among the soils, which mainly depends on other soil physico-chemical and biological properties of soil. Hence, it is difficult to compare the data of survival time of O157:H7 from other studies with the present data because of difference in soil properties and experimental set-ups.

Our results indicated that the survival of O157 in four different tropical soils and in cocopeat depends on physico-chemical and biological properties including pH, nitrogen, potassium, microbial biomass carbon and dehydrogenase activity. The principal component analysis of assessed variables showed a substantial difference in *E*. *coli* O157:H7 survival time between the soils that are differed in those soil properties. Interestingly, the total organic carbon did not correlate the survival of *E*. *coli* which recorded less contribution to the survival variability of strains recorded among the tested soils. Franz et al. [17] explained the O157 survival by dissolved organic carbon and microbial biomass carbon. In the present study, the microbial biomass carbon is well-correlated (positively) to the survival of O157-TNAU. Soil dehydrogenase, the functions of total range of oxidative activity and viable microbial populations, serves as a good indicator of soil microbial activity. It is also an indirect indicative of soil available nutrients for microbial processes. More of active nutrients and carbon if present in the soil obviously reported to have high dehydrogenase activity. In the present study, the soils with higher dehydrogenase (red lateritic soil and tropical latosol) recorded longer-survival of O157 than those with low dehydrogenase (wetland soil and black cotton soil). Van Elsas et al. [30] reported that the diversity of microbial community of soil is inversely proportional to the survival of O157:H7. Because of competition for nutrient and niche space as well as predation, the survival of introduced O157:H7 can be affected due to native microbial communities. It is generally accepted that more dehydrogenase means more diversified microbial communities [65], but still long survival of O157 was reported in those soils needs further investigations. Among the other nutrient properties assessed, nitrogen and potassium availability positively correlated with the survival of O157 with insignificance to phosphorus. More the nutrients recorded longer the survival of O157 is in accordance with the results of Ma et al. [55].

The soil pH influences significantly to *ttd* of *E*. *coli*. The study comparing the acidic and neutral soils of China claimed that the acidic soils (between 5.1 to 4.6) had very shorter *ttd* (about 7 days), while the near neutral soils (6.5 to 7.2) had longer *ttd* (about 30 days) [20]. In the present study, the pH of the soils is slightly acidic (5.9 to 6.05) and alkaline (8.4 to 8.7) nature and among which the alkaline soils had quick decline of O157 than slightly acidic condition. This result suggests that near neural pH is most conducive condition for survival of *E*. *coli* than alkaline and acidic pH. There may be several reasons, why soil pH had significant role in the survival of O157. At near neutral pH, any soil bacteria can adapt to the soil environment and can freely present in the solution. When the pH falls (below 5.0), the cells intended for sorption on the minerals and thereby decline their population [66]. Additionally, low available P, organic N, Al and Mn toxicity are the indirect effects cause survival and activity of O157:H7 in acid soils [20, 67]. On the other hand, at alkaline pH (above 8.0), the Ca^2+^ and Mg^2+^ ions cause significant reduction of O157, which was evident by recent work [68]. In the present study, the reduction of O157:H7 in alkaline soil and longer-persistence in near neutral soils are in accordance with these findings.

In contrast to four agricultural soils, cocopeat, a common root medium for vegetable nursery and medium for soil-less agriculture in greenhouses was also assessed for the O157 survival. The results suggest that O157 can survive equally in cocopeat as compared to agricultural soils. When compare to O157 (70 days of *ttd*), MTCC433 and DH5α had shorter survival (40 days of *ttd*) needs further investigations. Cocopeat is one of the best media widely adopted in Europe and several Asian countries to grow vegetables under controlled condition. Persistence of O157 reported through this investigation will be more useful to develop some simple strategies to avoid this food borne pathogen invasion in to vegetables.

In the present study, the survival data of all the three strains were successfully modeled by the Double Weibull model. This model is based on the assumption that there are two subpopulations present in the introduced strain and they differ in level of resistance to stress and survival of both the subpopulations follow Weibull distribution. The subpopulation with smaller δ perishes quickly compared to larger δ. In the present investigation, it was reported as δ1<δ2 for all the soils and for all the strains, suggests that the survival behavior of subpopulations differ significantly. However, O157:H7 had less difference between δ1 and δ2 (mean of about 15 days for all the soils) compare to DH5α and MTCC433 (about 21 days for each). With reference to soil, S1 and S3 had very less values of δ suggesting that both subpopulations are reduced more quickly in those soils, whereas the other soils (S2 and S4) had higher δ values, will take longer time to perish. These modeling parameters are well-fit to our results and earlier works ([20, 21, 55]. The *ttd* calculated through the Double Weibull model also revealed that the soils with high MBC and dehydrogenase had longer survival than those soils with less value, which are in accordance with the earlier studies [18, 24, 55]. This was further evident from our PCA results correlating the model parameters with observed soil variables. The *ttd*, t_4D_, δ1 and δ2 of modeling data were significantly correlated with MBC and dehydrogenase activity of soils (p = 0.001; S2, S3 and S4 Tables). Therefore the abundance of available carbon (reflected by MBC of present study) in soil can provide more nutrients, niche for colonization of O157:H7 in soil with decreased competition with indigenous population, slow down the decline of *E*. *coli* O157:H7.

The survival of O157:H7 in different agro-ecological soils across the world was well-documented [69, 70]. Most of the studies compared soil properties, organic amendments and environmental conditions on the fate of O157:H7. Few studies also compared the variants of O157:H7 on the survival under controlled [20, 21] and field conditions [9, 55]. The result clearly suggests that no significant difference was noticed between *stx* mutants and wild O157:H7 strain [55]. Several works comparing other STEC strains (non-O157:H7 strains) with O157:H7 also suggest that the difference of survival pattern among these strains was trivial [21]. However, no study has done so far by comparing natural non-pathogenic isolates and genetically engineered strains with O157:H7. In the present study, a natural, non-pathogenic human intestine isolate (MTCC433) and genetically engineered strain (DH5α) were compared with O157 for the soil persistence. The results clearly showed that the survival of O157 (TNAU isolate) and MTCC433 had a similar pattern, while DH5α had less persistence. Hence it is revealed that the natural isolates of *E*. *coli*, either pathogenic or non-pathogenic exhibit similar pattern of survival, while the genetically modified lab strains such as DH5α had much shorter survival in soil. The loss of competitiveness for nutrients and niche due to genetic engineering may be the reason for less persistence in all the soils and cocopeat tested, which needs further investigation.

## Conclusions

The present study for the first time showed that the genetically engineered *E*. *coli* strain (DH5α) perished quicker than non-pathogenic (MTCC433) and pathogenic (O157:TNAU) natural isolates. The soil incubation studies conducted under controlled condition showed significant difference in the survival rate of O157 and other strains among the four different tropical soils tested. O157 had the survival period ranged from 42 to 62 days in tropical soils tested and in cocopeat, its survival is about 69 days. The physico-chemical and biological properties which control the survival of O157 are pH, available N, K, microbial biomass carbon and dehydrogenase activity. These results are important with respect to microbial safety in vegetable production. Our results indicate that if O157:H7 contaminated the agricultural soils by any means; their survival is much shorter in these tropical soils compared to temperate soils. Bright sunshine prevailing in this region (About 12 hours) is another detrimental factor for pathogen survival in soil, which was not included in the present study, may still reduce the survival of O157:H7 under natural conditions. Hence, more work has to be done to assess the fate of O157:H7 under real field conditions of tropical soils; how different organic manures alter the survival of this pathogen in soil and is there any translocation of pathogen takes place from soil to vegetables when grown in contaminated soils.

## Supporting Information

S1 Table Loading values and per cent contribution of assessed soil variables (physico-chemical, microbiological and enzymatic activities) and survival parameters of *E*. *coli* strains on the axis identified by the principal component analysis. (DOC)

S2 Table Correlation matrix (Pearson (n-1)) of assessed soil variables and survival parameters of *E*. *coli* (DH5α). (DOC)

S3 Table Correlation matrix (Pearson (n-1)) of assessed soil variables and survival parameters of *E*. *coli* (MTCC433). (DOC)

S4 Table Correlation matrix (Pearson (n-1)) of assessed soil variables and survival parameters of *E*. *coli* (O157:TNAU). (DOC)
