# Supplementary material for: Persistence of Pathogenic and Non-Pathogenic Escherichia coli Strains in Various Tropical Agricultural Soils of India
Source: PLoS One. 2015 Jun 23;10(6):e0130038. doi: 10.1371/journal.pone.0130038 (PMC4477969; doi:10.1371/journal.pone.0130038)
Supplement: S1 Table — (DOC) [file pone.0130038.s001.doc]

**S1 Table.** Loading values and per cent contribution of assessed soil variables (physico-chemical, microbiological and enzymatic activities) and survival parameters of *E. coli* strains on the axis identified by the principal component analysis

| Variables | DH5α | | | | MTCC433 | | | | O157-TNAU | | | |
| --- | --- | --- | --- | --- | --- | --- | --- | --- | --- | --- | --- | --- |
|  | PC1 | | PC2 | | PC1 | | PC2 | | PC1 | | PC2 | |
|  | Loading value | % | Loading value | % | Loading value | % | Loading value | % | Loading value | % | Loading value | % |
| MP | 0.53 | 3.71 | 0.23 | 1.20 | -0.82 | 8.28 | 0.16 | 0.52 | -0.40 | 2.59 | 0.89 | 14.19 |
| DR | 0.18 | 0.45 | -0.40 | 3.60 | 0.38 | 1.76 | -0.18 | 0.65 | -0.12 | 0.24 | -0.34 | 2.14 |
| %R | -0.36 | 1.78 | 0.40 | 3.56 | -0.48 | 2.77 | 0.72 | 10.95 | 0.52 | 4.29 | 0.39 | 2.72 |
| tdd | 0.87 | 10.11 | -0.27 | 1.59 | -0.79 | 7.73 | -0.55 | 6.23 | -0.72 | 8.27 | -0.52 | 4.92 |
| t4D | 0.83 | 9.20 | -0.38 | 3.18 | -0.74 | 6.76 | -0.64 | 8.50 | -0.68 | 7.40 | -0.59 | 6.29 |
| delta1 | 0.97 | 12.52 | 0.17 | 0.62 | -0.91 | 10.12 | -0.20 | 0.83 | -0.74 | 8.66 | -0.17 | 0.51 |
| delta2 | 0.95 | 12.03 | -0.22 | 1.10 | -0.96 | 11.23 | 0.00 | 0.00 | -0.75 | 9.01 | 0.09 | 0.14 |
| pH | -0.86 | 9.78 | -0.51 | 5.77 | 0.96 | 11.28 | -0.25 | 1.29 | 0.84 | 11.15 | -0.53 | 5.12 |
| EC | -0.08 | 0.09 | 0.95 | 19.86 | -0.17 | 0.37 | 0.96 | 19.39 | 0.12 | 0.23 | 0.97 | 16.79 |
| MBC | 0.84 | 9.52 | -0.47 | 4.92 | -0.67 | 5.51 | -0.68 | 9.56 | -0.83 | 10.93 | -0.43 | 3.33 |
| SOC | -0.09 | 0.10 | 0.95 | 19.74 | -0.17 | 0.34 | 0.96 | 19.50 | 0.13 | 0.26 | 0.96 | 16.62 |
| DHA | 0.80 | 8.56 | -0.54 | 6.51 | -0.61 | 4.55 | -0.74 | 11.54 | -0.81 | 10.42 | -0.52 | 4.96 |
| N | 0.74 | 7.40 | 0.63 | 8.65 | -0.90 | 9.92 | 0.38 | 2.95 | -0.74 | 8.64 | 0.64 | 7.29 |
| P | 0.46 | 2.81 | 0.77 | 13.11 | -0.69 | 5.85 | 0.54 | 6.18 | -0.48 | 3.74 | 0.73 | 9.56 |
| K | -0.81 | 8.79 | -0.55 | 6.57 | 0.93 | 10.60 | -0.27 | 1.48 | 0.81 | 10.52 | -0.55 | 5.37 |
| TCB | -0.48 | 3.14 | 0.02 | 0.01 | 0.49 | 2.93 | 0.14 | 0.42 | 0.48 | 3.66 | -0.06 | 0.07 |
| Eigen value | 7.48 | | 4.53 | | 8.15 | | 4.77 | | 6.28 | | 5.56 | |
| Variability (%) | 46.78 | | 28.29 | | 50.96 | | 29.84 | | 39.24 | | 34.76 | |
| Cumulative % | 46.78 | | 75.06 | | 50.96 | | 80.80 | | 39.24 | | 74.00 | |

% refers per cent contribution of each variable to respective PC. Values in bold explained >50% contribution to the significant component.MP –Mean population at respective days interval; DR – Decimal reduction rate; % R – Mean per cent reduction of population; tdd – Time to reach the detection limit; t_4D_, time (days) to attain a 4 log reduction; delta1 - time (days) for first decimal reduction of subpopulation 1; delta2 - time (days) for first decimal reduction of subpopulation 2; EC – Electrical conductivity; MBC – Microbial biomass carbon; SOC – Soil organic carbon; DHA – Dehydrogenase activity; TCB – Total culturable bacterial counts.
