# Supplementary material for: Persistence of Pathogenic and Non-Pathogenic Escherichia coli Strains in Various Tropical Agricultural Soils of India
Source: PLoS One. 2015 Jun 23;10(6):e0130038. doi: 10.1371/journal.pone.0130038 (PMC4477969; doi:10.1371/journal.pone.0130038)
Supplement: S3 Table — (DOC) [file pone.0130038.s003.doc]

**S3 Table.** Correlation matrix (Pearson (n-1)) of assessed soil variables and survival parameters of *E. coli* (MTCC433)

| Variables | MP | DR | %R | tdd | t4D | delta1 | delta2 | pH | EC | MBC | SOC | DHA | N | P | K |
| --- | --- | --- | --- | --- | --- | --- | --- | --- | --- | --- | --- | --- | --- | --- | --- |
| DR | -0.11 |  |  |  |  |  |  |  |  |  |  |  |  |  |  |
| %R | 0.35 | -0.57* |  |  |  |  |  |  |  |  |  |  |  |  |  |
| tdd | 0.61* | -0.13 | -0.05 |  |  |  |  |  |  |  |  |  |  |  |  |
| t4D | 0.47 | -0.18 | -0.07 | 0.97** |  |  |  |  |  |  |  |  |  |  |  |
| delta1 | 0.73** | -0.29 | 0.22 | 0.77** | 0.74** |  |  |  |  |  |  |  |  |  |  |
| delta2 | 0.73** | -0.39 | 0.45 | 0.70** | 0.69** | 0.96** |  |  |  |  |  |  |  |  |  |
| pH | -0.81** | 0.36 | -0.62* | -0.61* | -0.55* | -0.85** | -0.95** |  |  |  |  |  |  |  |  |
| EC | 0.31 | -0.15 | 0.73** | -0.42 | -0.52* | 0.01 | 0.19 | -0.42 |  |  |  |  |  |  |  |
| MBC | 0.36 | -0.25 | -0.08 | 0.84** | 0.92** | 0.77** | 0.70** | -0.49 | -0.53* |  |  |  |  |  |  |
| SOC | 0.32 | -0.14 | 0.74** | -0.42 | -0.52* | 0.02 | 0.19 | -0.42 | 0.99** | -0.53* |  |  |  |  |  |
| DHA | 0.37 | -0.14 | -0.23 | 0.86** | 0.90** | 0.72** | 0.60* | -0.40 | -0.59* | 0.93** | -0.60* |  |  |  |  |
| N | 0.77** | -0.36 | 0.71** | 0.52* | 0.45 | 0.69** | 0.84** | -0.95** | 0.52* | 0.35 | 0.50 | 0.28 |  |  |  |
| P | 0.72** | -0.27 | 0.67** | 0.36 | 0.23 | 0.39 | 0.55* | -0.78** | 0.59* | 0.01 | 0.58* | -0.02 | 0.86** |  |  |
| K | -0.77** | 0.39 | -0.58* | -0.61* | -0.53* | -0.80** | -0.89** | 0.96** | -0.42 | -0.42 | -0.41 | -0.36 | -0.94** | -0.82** |  |
| TCB | -0.46 | -0.07 | -0.16 | -0.51* | -0.44 | -0.39 | -0.37 | 0.39 | 0.01 | -0.36 | 0.06 | -0.30 | -0.38 | -0.24 | 0.36 |

MP –Mean population at respective days interval; DR – Decimal reduction rate; % R – Mean per cent reduction of population; tdd – Time to reach the detection limit; t_4D_, time (days) to attain a 4 log reduction; delta1 - time (days) for first decimal reduction of subpopulation 1; delta2 - time (days) for first decimal reduction of subpopulation 2; EC – Electrical conductivity; MBC – Microbial biomass carbon; SOC – Soil organic carbon; DHA – Dehydrogenase activity; TCB – Total culturable bacterial counts.
